# tagtango: an application to compare single-cell annotations

**DOI:** 10.1093/bioinformatics/btaf012

**Published:** 2025-01-11

**Authors:** Bernat Bramon Mora, Helen Lindsay, Antonin Thiébaut, Kenneth D Stuart, Raphael Gottardo

**Affiliations:** Biomedical Data Science Center, Lausanne University Hospital, Vaud 1005, Switzerland; Biomedical Data Science Center, University of Lausanne, Vaud 1015, Switzerland; Swiss Institute of Bioinformatics, Vaud 1015, Switzerland; Biomedical Data Science Center, Lausanne University Hospital, Vaud 1005, Switzerland; Biomedical Data Science Center, University of Lausanne, Vaud 1015, Switzerland; Swiss Institute of Bioinformatics, Vaud 1015, Switzerland; Biomedical Data Science Center, Lausanne University Hospital, Vaud 1005, Switzerland; Biomedical Data Science Center, University of Lausanne, Vaud 1015, Switzerland; Swiss Institute of Bioinformatics, Vaud 1015, Switzerland; Center for Global Infectious Disease Research, Seattle Children’s Hospital, WA 98105, United States; Biomedical Data Science Center, Lausanne University Hospital, Vaud 1005, Switzerland; Biomedical Data Science Center, University of Lausanne, Vaud 1015, Switzerland; Swiss Institute of Bioinformatics, Vaud 1015, Switzerland; School of Life Sciences, EPFL - Swiss Federal Technology Institute of Lausanne, Lausanne, Vaud 1015, Switzerland

## Abstract

**Summary:**

In this article, we present tagtango, an innovative R package and web application designed for robust and intuitive comparison of single-cell clusters and annotations. It offers an interactive platform that simplifies the exploration of differences and similarities among different clustering and annotation methods. Leveraging single-cell data analysis and different visualizations, it allows researchers to dissect the underlying biological differences across groups. tagtango is a user-friendly application that is portable and works seamlessly across multiple operating systems.

**Availability and implementation:**

tagtango is freely available at https://github.com/bernibra/tagtango as an R package as well as an online web service at https://tagtango.unil.ch.

## 1 Introduction

Two integral components of single-cell data analysis are cell clustering and annotation (Kiselev *et al.* 2019). They are central to most downstream analyses (e.g. differential expression analyses; Finak *et al.* 2015), allowing us to study relevant biological processes such as transcriptional changes and cell/gene interactions (Kumar *et al.* 2018, van Dijk *et al.* 2018). This has led to the development of a diverse collection of clustering and annotation methods to automate the identification of cell populations (de Kanter *et al.* 2019, Alquicira-Hernandez *et al.* 2019, Hao *et al.* 2021). This diversity of methods and granularity of annotations, however, in combination with the heterogeneous nature of biological data, can lead to large variability in the identification of phenotypes (Ziegenhain *et al.* 2017). The emergence of multimodal single-cell sequencing technologies—e.g. surface protein expression (Stoeckius *et al.* 2017), DNA methylation (Gaiti *et al.* 2019), chromatin accessibility (Cao *et al.* 2018), and spatial transcriptomics (Ståhl *et al.* 2016)—has opened the door to applying such annotation methods to different data modalities, adding even more complexity to the identification and classification of cell types. Indeed, new data modalities could provide additional resolution to identify cell populations that cannot be distinguished solely based on RNA expression (Ding *et al.* 2020). Therefore, we need tools that help us compare annotations and clusters, untangling differences and similarities across populations. Here, we introduce tagtango, an R package and user-friendly web application designed for comparing single-cell annotations and clusters. This tool offers an easy way to shed light on inconsistencies present across various cell identification methods and better understand whether the variations across annotations contain relevant biological information or are the product of the idiosyncrasies of different data types and methods.

## 2 Materials and methods

**Figure 1.**
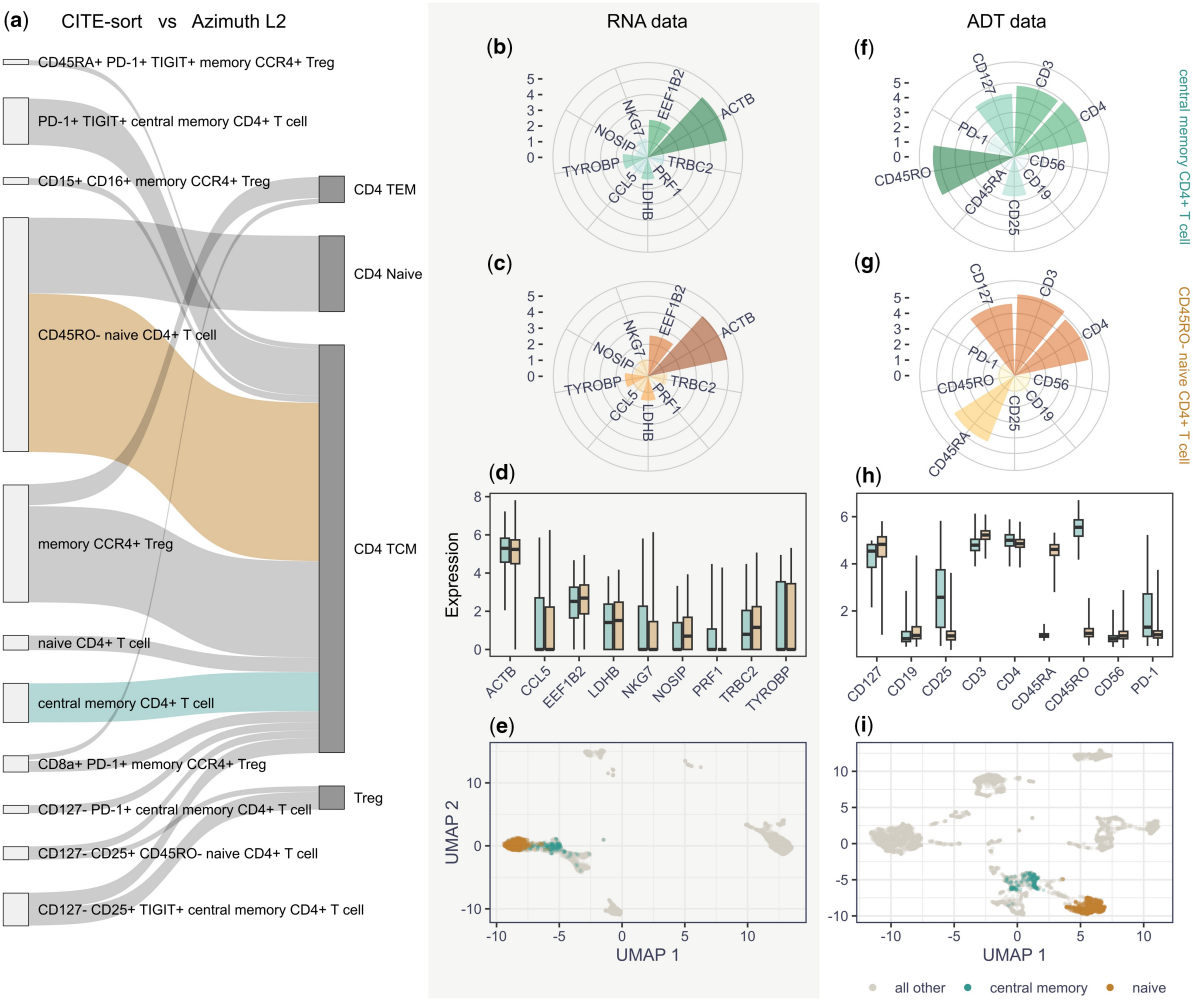
Overview of the annotation comparison performed by tagtango. Panel (a) displays a Sankey diagram comparing the annotations performed by CITE-sort and Azimuth’s level 2 (i.e. ‘celltype.l2’). The diagram was filtered using tagtango to only include cells annotated as ‘CD4+ T-cells’ by Azimuth’s main cell type classification (i.e. ‘celltype.l1’) and links containing at least 20 cells. The coloured links in the diagram indicate the cell populations selected for deeper analysis. Panels (b) and (f) display rose plots of the normalized expression for the genes and protein markers deemed most relevant in the selected cell population ‘central memory CD4+ T cell’. Panels (c) and (g) display the same for the selected cell population ‘CD45RO- naive CD4+ T cell’. Panels (d) and (h) display a direct comparison between the ADT and RNA marker normalized expression for the two selected cell populations, including only those relevant markers. The colours of the bars match those of the selected links in panel (a). Panels (e) and (i) present the UMAP representation of all cells calculated using all markers for the ADT and RNA expression data, respectively. The colours of the points match those of the selected links in panel (a).

tagtango is a software package to compare multiple annotations associated to a single-cell dataset. The input data can be provided in standard Bioconductor formats (Gentleman *et al.* 2004): a ‘MultiAssayExperiment’ object stored as an R Data Serialization (RDS) file (Ramos *et al.* 2017); a ‘SingleCellExperiment’ object stored as an RDS file (Amezquita *et al.* 2020); or a data frame as an RDS, comma-separated values, or tab-separated values file. The core functionality of tagtango centres around running a web application that displays a detailed comparison of annotations generated by different methods and data types. That is, given an input data object containing a normalized expression matrix and a set of annotations, tagtango will study the differences in marker expression across cell populations (see Supplementary Material for data specifications). This application also allows the user to hone in on relevant differences across annotation strategies by strategically grouping populations across main cell types or other variables, as well as filtering out noise or inconsistencies across them. Notably, tagtango allows users to export all results and figures as R code (see Fig. 1), ensuring reproducibility for every visualization produced by the tool.

Given two cell populations of interest, tagtango uses different strategies to select the relevant markers displayed in the visualizations. When working with low-dimensional data modalities, such as antibody-derived tags (ADT) expression data, tagtango identifies relevant protein markers by studying differences in average expression between populations, allowing the users to also specify other required markers in the visualizations. In contrast, when working with RNA expression data (or other high-dimensional data modalities; i.e. >1000 features), tagtango first uses the function ‘scoreMarkers’ from the R package ‘scran’ (Lun *et al.* 2016) to quantify the expression differences across every possible annotation or cluster, computing summary scores for each marker in each group of cells. Then, it selects the 10 most upregulated markers for every group using the median Cohen’s d. Finally, it identifies the markers displayed in the visualization by studying the average expression differences between any selected populations, allowing the users again to include any other upregulated marker across possible annotations or clusters. Notice that, alternatively, one can provide filtered RNA expression data including only gene markers of interest, thus bypassing the need for the pre-selection using ‘scran’ and speeding up the analyses.

The implementation of tagtango involves an R package and a shiny application, which use Javascript and R libraries such as ‘networkD3’ (Allaire *et al.* 2017) and ‘ggplot2’ (Wickham 2016) to generate the visualizations. The software, distributed under the MIT license, is cross-platform and freely available at https://github.com/bernibra/tagtango. The accompanying web application is hosted at https://tagtango.unil.ch, though, for cost reasons, it is currently restricted to smaller tests (i.e. approximately >10 000 cells and <2000 features). In terms of resource requirements, the test dataset (7472 cells) requires approximately 840 MB of memory to perform operations in R. Running tagtango on this dataset using the low-dimensional data approach (fewer than 2000 features) requires 1 CPU and approximately 1010 MB of RAM. For higher-dimensional data modalities, the internal use of ‘scoreMarkers’ by tagtango increases the RAM consumption up to 4.2GB for the same dataset (using 33 538 features). Both resource and time scalability will depend on the number of unique annotations or clusters and the use of ‘scoreMarkers’, which has been benchmarked in the past using multiple datasets (Pullin and McCarthy 2024).

## 3 Usage scenario: ADT vs RNA-based annotations

To illustrate the functionality of tagtango, we used the preprocessed and annotated dataset provided with the R package and web application. This is a ‘MultiAssayExperiment’ with peripheral blood mononuclear cells from a healthy donor, stained with TotalSeq-B antibodies (10x Genomics 2018). To normalize the RNA expression matrix, we used the ‘logNormCounts’ function from the R package ‘scuttle’ (McCarthy *et al.* 2017), computing therefore the log-transformed normalized expression values. Similarly, we normalized the ADT expression data using the R package ‘ADTnorm’ (Zheng *et al.* 2022), a tool specifically designed for the normalization of CITE-seq data. The dataset was annotated using different algorithms. In particular, we annotated cells using RNA expression data with Azimuth (Hao *et al.* 2021), and the ADT with CITE-sort (Lian *et al.* 2020).

Using tagtango, we compared the two sets of annotations. First, we grouped cells based on their main cell-type classification (Azimuth level 1) and selected CD4 T-cells as our main focus. With this information, tagtango produces a Sankey diagram, where nodes on each side represent the different annotations and links characterize their associations. Figure 1a shows the resulting diagram when filtering out links and nodes containing <20 cells (note that this parameter can be adjusted in the application).

The Sankey diagram allows the user to select any pair of nodes or links and compare the corresponding cell populations. In Fig. 1a, we selected two populations: cells annotated as ‘central memory CD4+ T cell’ by CITE-sort, and cells annotated as ‘CD45RO- naive CD4+ T cell’ by CITE-sort. Notice that these two populations appear somewhat inconsistent with each other, as the CITE-sort annotations differentiated two cell types while Azimuth classified them both as central memory CD4+ T-cells (i.e. ‘CD4 TCM’). Using the normalized ADT and RNA expression data, tagtango produced several visualizations to illustrate their differences. While the RNA visualizations found similar marker expression levels between annotations (Fig. 1b–e), the ADT visualizations identified markers CD45RA and CD45RO as the primary responsible for such inconsistencies (Fig. 1f–i). This seems to support the distinction made by CITE-sort, as Naive CD4+ T cells should be characterized by the expression of CD45RA, which indicate cells that have not encountered antigens. Likewise, memory CD4+ T cells should exhibit CD45RO expression, indicating a history of antigen exposure and differentiation (Henson *et al.* 2012). Overall, this highlights the potential challenge that annotations relying on RNA expression may encounter in distinguishing between various isoforms of a given protein; in this case, two isoforms CD45RA and CD45RO resulting from the alternative splicing of the PTPRC gene. In contrast, however, both the RNA and ADT UMAP representations (Fig. 1e and i) seem to separate well the two cell populations, showing the cumulative effect of differences in the expression across genes.

## 4 Conclusion

As the landscape of single-cell technologies and annotation methods continues to grow, we need tools to compare and interpret annotations and clusters identified by different algorithms and across data modalities (Freytag *et al.* 2018, Xu *et al.* 2022). tagtango is an R package and web application developed to facilitate such comparisons. Taking a single-cell dataset as input, tagtango facilitates the analysis and synthesis of the differences and similarities across any set of annotations by producing visualizations comparing specific cell populations. In this work, we showcased its utility by comparing annotations produced using RNA and ADT expression data in a CITE-seq dataset. In particular, we were able to key in on specific inconsistencies across methods, shedding light on the potential pitfalls of each annotation strategy. Moreover, we provided additional examples of how tagtango can be used across data modalities (see the ‘Supplementary usage scenario: comparing spatial transcriptomics annotations’ section of the Supplementary Information) and for any type of annotation (including samples information as shown in the ‘Supplementary usage scenario: understanding batch effects’ section of the Supplementary Information). Likewise, we provided an example of cross-dataset comparisons using independent single-cell experiments with tagtango (see the ‘Supplementary usage scenario: comparing single-cell datasets’ section of the Supplementary Information). Overall, we believe that this new tool could be of great use for the single-cell community, enabling the comparison of annotations to become a routine part of an analysis, even for non-expert users.

## Supplementary Material

btaf012_Supplementary_Data

## Supplementary data

Supplementary data are available in the R package at https://github.com/bernibra/tagtango and in the article's online supplementary material.
