# Supplementary material for: tagtango: an application to compare single-cell annotations
Source: Bioinformatics. 2025 Jan 11;41(2):btaf012. doi: 10.1093/bioinformatics/btaf012 (PMC11814489; doi:10.1093/bioinformatics/btaf012)
Supplement: btaf012_Supplementary_Data [file btaf012_supplementary_data.zip › 510fc_tagtango_1.0.0.pdf]

Package ‘tagtango’

December 11, 2024

Title A web app for comparing cell annotations or clusterings

Version 1.0.0

Description The tagtango app is flexible in handling various data types. For now, however, it only accepts input in the form of either a MultiAssayExperiment object stored as an RDS file, a SingleCellExperiment object stored as an RDS file, or a data.frame stored as an RDS, CSV, or TSV file.

License MIT + file LICENSE

Imports aricode,
colorspace,
config (>= 0.3.2),
dplyr,
ggplot2,
ggtext,
golem (>= 0.4.1),
htmltools,
htmlwidgets,
magrittr,
Matrix,
methods,
MultiAssayExperiment,
networkD3,
rlang,
scran,
shiny (>= 1.7.5.1),
shinyalert,
shinyjs,
shinyWidgets,
SingleCellExperiment,
spelling,
stats,
stringr,
SummarizedExperiment

Encoding UTF-8

LazyData false

RoxygenNote 7.3.2

Suggests knitr,
rmarkdown,
testthat (>= 3.0.0)

Config/testthat/edition 3

Language en-US

VignetteBuilder knitr

Depends R (>= 2.10)

Contents

bar_diff 2
process_data 3
rose_plot 4
run_app 5
scatter_plot 6
test_data 6

Index **8**

`bar_diff` *Generate a ggplot bar plot with marker differences*

Description

A function to generate a barlot as 'tagtango' does.

Usage

```
bar_diff(
  norm,
  data,
  first_selection,
  second_selection,
  n_bars = 10,
  valley = NULL,
  palette = "BrBG",
  quant = NULL
)
```

Arguments

`norm`	normalized expression data with cells as rows and columns as markers/genes (i.e. function 'process_data()' should provide this under the attribute 'data')
`data`	data.frame with the different annotations, where each row represent a cell. The rownames should match those of 'norm' (i.e. function 'process_data()' should provide this under the attribute 'network').
`first_selection`	boolean array with first selection cells as TRUE values, for all cells in 'data'.
`second_selection`	boolean array with second selection cells as TRUE values, for all cells in 'data'.
`n_bars`	if an integer value, it defines the number of bars of the plot, selecting those that are most "relevant". If an array with marker names, it uses those.
`valley`	the value in 'norm' corresponding to the valley separating positive and negative peak for CITE-seq data.

`palette`	color palette, default "BrBG"
`quant`	position of positive and negative peak for normalized data.

Value

returns a list containing a ggplot object and the list of markers selected and used.

---

process_data — *Process data as the shiny application*

Description

A utility function for reading a processing the data as 'tagtango' does.

Usage

```
process_data(
  filename,
  data_type,
  left,
  right,
  pc1_axis1 = NULL,
  pc1_axis2 = NULL,
  pc2_axis1 = NULL,
  pc2_axis2 = NULL,
  filter_variable = NULL,
  filter_values = NULL,
  grouping_variable = NULL,
  grouping_values = NULL,
  min_counts = NULL,
  dimension = NULL,
  input_data = NULL
)
```

Arguments

`filename`	path to data file
`data_type`	experiment used in the MultiAssayExperiment or data contained in logcounts in SingleCellExperiment
`left`	annotation in the left of the diagram
`right`	annotation in the right of the diagram
`pc1_axis1`	first axis of first dimension reduction space
`pc1_axis2`	second axis of first dimension reduction space
`pc2_axis1`	first axis of second dimension reduction space
`pc2_axis2`	second axis of second dimension reduction space
`filter_variable`	filtering variable found in 'dat'
`filter_values`	values in 'filter_variable' that need to be excluded

`grouping_variable`
: grouping variable found in 'dat'

`grouping_values`
: values in 'grouping_variable' that need to be filtered

`min_counts`
: minimum number of cells in a link for this to be displayed

`dimension`
: internal variable to determine whether the data is a data frame (=0), a low-dimension dataset (=1), or a high-dimension dataset (=2).

`input_data`
: object inputed directly.

Value

Returns a list containing two attributes: data and network. The first contains the data filtered according to the grouping and filtering values. The second, contains the data organized in a manner that is readible by the sankeyNetwork.

---

rose_plot *Generate a ggplot rose plot with marker expression*

Description

A function to generate a rose plot as 'tagtango' does.

Usage

```
rose_plot(
  norm,
  data,
  selected,
  n_petals = 10,
  title = NULL,
  valley = NULL,
  palette = "RdYlGn",
  quant = c(1, 6),
  ...
)
```

Arguments

`norm`
: normalized expression data with cells as rows and columns as markers/genes (i.e. function 'process_data()' should provide this under the attribute 'data')

`data`
: data.frame with the different annotations, where each row represent a cell. The rownames should match those of 'norm' (i.e. function 'process_data()' should provide this under the attribute 'network').

`selected`
: boolean array with selected cells as TRUE values, for all cells in 'data'.

`n_petals`
: if an integer value, it defines the number of petals of the rose plot, selecting those that are most "relevant". If an array with marker names, it uses those.

`title`
: main plot title. Default 'NULL'.

`valley`
: the value in 'norm' corresponding to the valley separating positive and negative peak for CITE-seq data.

palette	color palette, default "RdYlGn".
quant	position of positive and negative peak for normalized data.
...	parameters passed to the underlying function.

Value

returns a ggplot object.

---

run_app *Run the Shiny Application*

Description

Run the Shiny Application

Usage

```
run_app(
  onStart = NULL,
  options = list(),
  enableBookmarking = NULL,
  uiPattern = "/",
  maxRequestSize = 3000 * 1024^2,
  input_data = NULL,
  ...
)
```

Arguments

onStart	A function that will be called before the app is actually run. This is only needed for shinyAppObj, since in the shinyAppDir case, a global.R file can be used for this purpose.
options	Named options that should be passed to the runApp call (these can be any of the following: "port", "launch.browser", "host", "quiet", "display.mode" and "test.mode"). You can also specify width and height parameters which provide a hint to the embedding environment about the ideal height/width for the app.
enableBookmarking	Can be one of "url", "server", or "disable". The default value, NULL, will respect the setting from any previous calls to enableBookmarking(). See enableBookmarking() for more information on bookmarking your app.
uiPattern	A regular expression that will be applied to each GET request to determine whether the ui should be used to handle the request. Note that the entire request path must match the regular expression in order for the match to be considered successful.
maxRequestSize	maximum file size allowed in the application, in bytes. Default is 3Gb. Increase at your own risk.
input_data	dataset added via the command line. This will skip the process of uploading data unless the data is malformatted.
...	arguments to pass to golem_opts. See '?golem::get_golem_options' for more details.

`scatter_plot` *Generate a ggplot scatter plot*

Description

A function to generate a scatter plot as 'tagtango' does.

Usage

```
scatter_plot(
  data,
  labels,
  values = c("a", "b"),
  title = "UMAP of the RNA data",
  xlabel = "first axis",
  ylabel = "second axis"
)
```

Arguments

`data`	data.frame with dimension reduction axes.
`labels`	array with labels for first (and potentially second selections), for all cells in 'data'.
`values`	boolean array with second selection cells as TRUE values, for all cells in 'data'.
`title`	boolean array with second selection cells as TRUE values, for all cells in 'data'.
`xlabel`	the text for the x axis.
`ylabel`	the text for the y axis.

Value

returns a ggplot object.

`test_data` *Test CITE-seq data*

Description

A subset of the 10x dataset: 10k PBMCs from a healthy donor - gene expression and cell surface protein, single cell gene expression (Single Cell Gene Expression Dataset by Cell Ranger 3.0.0) Peripheral blood mononuclear cells (PBMCs) from a healthy donor stained with TotalSeq-B antibodies.

Usage

```
data("test_data")
```

Format

'test_data' A SingleCellExperiment with 7472 cells, 17 protein markers, and 33538 gene IDs:

date November 19, 2018

Source

<https://support.10xgenomics.com/single-cell-gene-expression/datasets/3.0.0/pbmc_10k_protein_v3>

Index

∗ **datasets**
test_data, 6

bar_diff, 2

enableBookmarking(), *5*

process_data, 3

rose_plot, 4
run_app, 5

scatter_plot, 6

test_data, 6
