# Supplementary material for: tagtango: an application to compare single-cell annotations
Source: Bioinformatics. 2025 Jan 11;41(2):btaf012. doi: 10.1093/bioinformatics/btaf012 (PMC11814489; doi:10.1093/bioinformatics/btaf012)
Supplement: btaf012_Supplementary_Data [file btaf012_supplementary_data.zip › 0a9b0_supplementary_information.pdf]

Supplementary Information

tagtango: an application to compare single-cell annotations

Bernat Bramon Mora[1,2,3,*], Helen Lindsay[1,2,3], Antonin Thiébaut[1,2,3], Kenneth D Stuart[4], and Raphael Gottardo[1,2,3,*]

[1]Lausanne University Hospital

[2]University of Lausanne

[3]Swiss Institute of Bioinformatics, Lausanne, Switzerland

[4]Seattle Children's Hospital

[*]To whom correspondence should be addressed

Supplementary Methods

`tagtango` is a powerful web application and R package designed for the comprehensive analysis and comparison of multiple cell annotations performed on a single-cell dataset. Leveraging multiple data modalities, this tool allows you to untangle the differences and similarities of cell populations, effectively distinguishing real differences across cell annotations from background noise. The next sections describe the data specifications.

Input data requirements

`tagtango` is flexible in handling various data types. To date, it accepts input in the form of either a `MultiAssayExperiment` R object stored as an RDS file[18], a `SingleCellExperiment` R object stored as an RDS file[2], or an R data frame stored as an RDS, CSV, or TSV file.

`MultiAssayExperiment` *object*

Providing a `MultiAssayExperiment` object as input will allow you to study multiple data modalities simultaneously. However, there are certain criteria that needs to be met. First, the elements of the `ExperimentList` container should be `SingleCellExperiment` following the specifications stated in the next section. Second, cells in all elements of the `ExperimentList` should be the same (and have unique and matching names). Finally, different annotations should be stored as columns of the `colData` data frame within the object.

As test dataset, a preprocessed and annotated 10x dataset is provided with the package. This is a `MultiAssayExperiment` with Peripheral Blood Mononuclear Cells (PBMCs) from a healthy donor stained with a few TotalSeq-B antibodies[6], and is readily accessible via:

```
library(tagtango)
data(test_data)
test_data
```

Notice that the column and row names of each `SingleCellExperiment` in the corresponding `ExperimentList` object are not `NULL`, and that the different annotations are stored in:

```
MultiAssayExperiment::colData(test_sce)
```

`SingleCellExperiment` *object*

Providing a `SingleCellExperiment` object, one also needs to ensure that the data is formatted in a specific manner. First, data should be normalized and stored as a `logcounts` assay within the `SingleCellExperiment` object. For example, in the main text, CITE-seq data was normalized using the R package *ADTnorm*. Second, cell and marker names within the `SingleCellExperiment` object should be defined (ensure these are not set as `NULL`). Finally, different annotations should be stored as columns of the `colData` within the object. Likewise, the object can contain the principal components of the data calculated using different decomposition techniques in `reducedDims` (see Amezquita *et al.*[2] for further information). Notice that an example of a `SingleCellExperiment` that can be used as input by `tagtango` can be generated using:

```
test_data[["ADT"]]
```

Data frame object

Providing a data frame object is the simplest way to run `tagtango`. The application expects different annotations to be stored as columns of the data frame. An example of a data frame that can be used as input by `tagtango` can be generated as:

```
MultiAssayExperiment::colData(test_data)
```

Supplementary usage scenario: comparing spatial transcriptomics annotations

To highlight the versatility of `tagtango`, we tested our software on a different data modality. We used an annotated spatial transcriptomics dataset provided as part of the 'spatialLIBD' project[13,17]. The data was generated with 10x Genomics Visium platform and contain human brain tissue samples from three healthy donors. In particular, these are spatially adjacent replicates of human dorsolateral prefrontal cortex tissue. This dataset is interesting because it investigates the laminar structure of the brain cortex, providing several manual annotations of the different layers for the individual spots (considered as 'ground truth') as

well as several cluster-based annotations.

Following the input data requirements outlined above, we prepared the dataset with R to be studied with `tagtango`. First, we followed Pardo *et al.*[17], and downloaded the spatial transcriptomics dataset.

```
# Load libraries
library(spatialLIBD)
library(SingleCellExperiment)
library(scater)

# Download data
spe <- fetch_data(type = "spe")
```

The data comes in the form of a `SpatialExperiment` R object. We reshaped the data into a `SingleCellExperiment` object using

```
sce <- SingleCellExperiment(
         list( logcounts = logcounts(spe) ),
         colData = cbind( colData(spe), spatialCoords(spe) ),
         rowData = rowData(spe)
      )

sce <- runUMAP(sce, exprs_values = "logcounts")
```

Notice that the spatial coordinates of the spatial transcriptomics dataset were added as columns in the `colData` object (these could also be added as part of the `reducedDims` object), and that we calculated the UMAP decomposition using the R package 'scater'[14].

Finally, this object contains multiple samples for a spatial transcriptomics dataset; therefore, the column names are not unique. Likewise, row names (i.e. gene IDs) can also be more intuitive if we use the gene names as opposed to Ensembl names. To fix this, we used:

```
# Row names
rownames(sce) <- rowData(sce)$gene_name

# Fix col names (they were not unique)
colnames(sce) <- rownames(data.frame(colData(sce)))
```

Using `tagtango`, we then compared sets of annotations for this dataset. A first interesting

comparison is that between the consensus manual annotations and one of the best performing unsupervised clustering method used in Maynard *et al.*[13]. In particular, we focused on the differences between 'ground truth' annotations and annotations using highly variable genes from 'scran'[11], 50 PCs for dimension reduction, and spatial coordinates as features (i.e. method 'HVG_PCA_spatial' as described in Maynard *et al.*[13]). Supplementary Figure **S1** explores the comparison between spots manually labelled as White Matter (WM) but annotated as two different clusters in the unsupervised approach. Notice that we used `tagtango` to filter out the results, focusing on one sample (i.e. labelled as '151675') excluding non-tissue spots (i.e. classified as 'NA' in the manual annotations). This comparison identified key genes driving the distinction between sub-populations within the manually annotated WM region. These include known WM and L5 marker genes such as MBP, which, coupled with the fact that spots in cluster 4 did not intersect with spots manually labelled as L5 or L6, suggests additional modularity within the WM layer. Similarly, a marker gene for gray matter/neurons that was used to define the sample orientation by Maynard *et al.*[13], SNAP25 is also identified as relevant. Finally, MOBP, a gene identified as top 10th most variable markers across layers by the author but that our comparison suggests this also show some level of modularity within WM, was also selected as relevant.

Notice that there are multiple types of comparisons that could be performed with `tagtango`. For example, we could try to understand differences between the way the different co-authors of the study labelled the spots, annotations that are also provided with the R package. Likewise, we could focus on comparing full layers, reproducing the authors results and potentially identifying additional marker genes separating the cortex layers.

Supplementary usage scenario: understanding batch effects

Another interesting use case for `tagtango` is the identification of marker differences across batches. To illustrate this, we used the same spatial dataset described in the previous section to compare manually annotated brain layers to donor IDs. Supplementary Figure **S2** explores the differences between WM in donors 'Br8100' and 'Br5595'. In this case, we see strong differences in the normalized expression of several interesting genes. For example, we see large differences again for MBP, a marker gene that is central to the

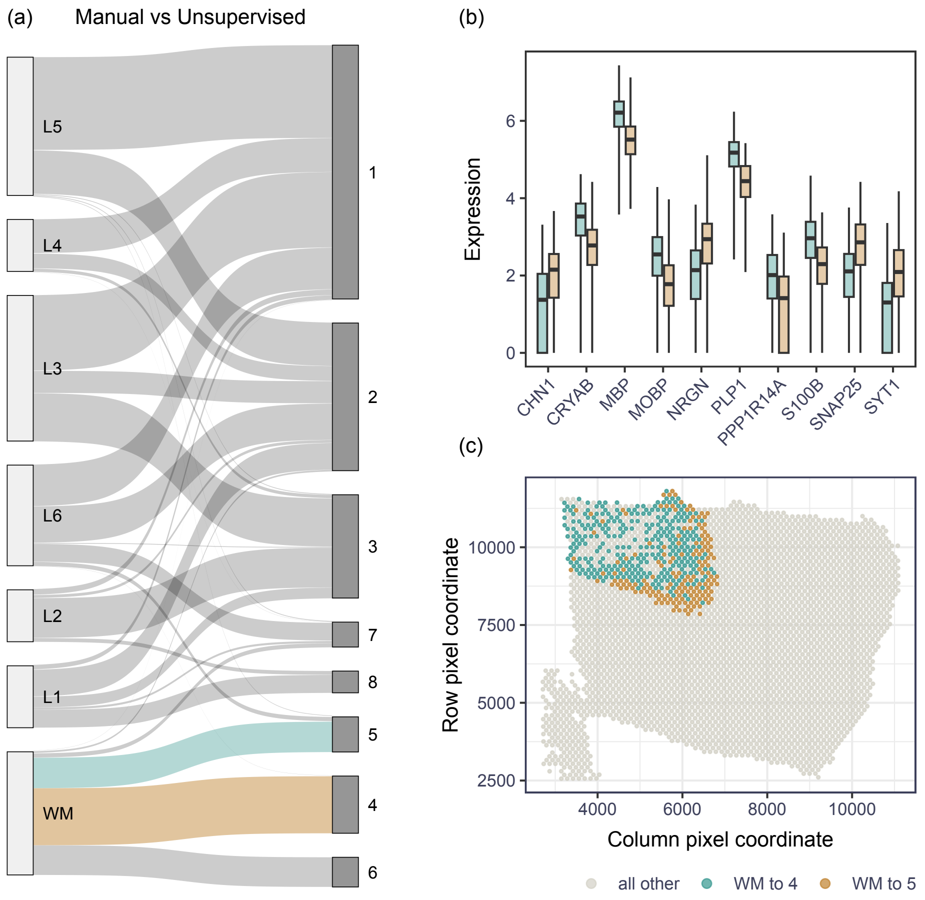

Supplementary Figure S1: Overview of the comparison between layer annotations in the brain cortex dataset. Panel (a) displays a Sankey diagram comparing the manual and unsupervised annotations. The diagram was filtered using `tagtango` to only include cells from sample '151675' and in-tissue spots. The coloured links in the diagram indicate the cell populations selected for White Matter (WM). Panel (b) displays a direct comparison of the normalized RNA expression for the two selected cell populations, including only markers selected as relevant. The colours of the bars match those of the selected links in panel (a). Panel (c) presents the spatial representation of all spots, where the colours of the points match those of the selected links in panel (a).

manual annotations. Likewise, we see batch effects in other notable genes such as CNP, identified by Zeng *et al.*[21] as brain cortex cell-type marker genes conserved between Human and Mouse. Finally, we see also differences for MOBP, highlighting this gene as not only variable across layers but also across batches.

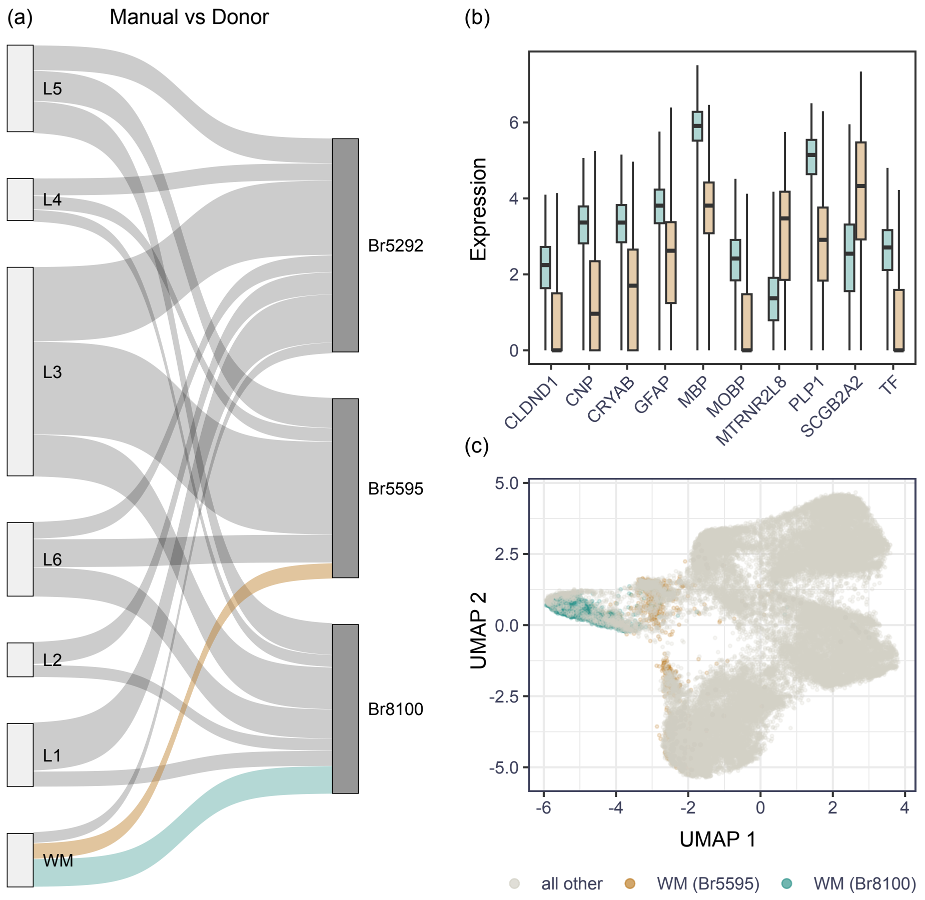

Supplementary Figure S2: Overview of the comparison between samples in the brain cortex dataset. Panel (a) displays a Sankey diagram comparing the manual annotations and donor ID. The diagram was filtered using `tagtango` to only include in-tissue spots. The coloured links in the diagram indicate the cell populations selected for deeper analysis. Panel (b) displays a direct comparison of the normalized RNA expression for the two selected cell populations, including only markers selected as relevant. The colours of the bars match those of the selected links in panel (a). Panel (c) presents the UMAP representation of the RNA expression for all spots, where the colours of the points match those of the selected links in panel (a).

Supplementary usage scenario: comparing single-cell datasets

As the final usage case scenario, we showcased how `tagtango` can be used to compare datasets. To do so, we used two independent 10x datasets: PBMCs from a healthy donor obtained by 10x Genomics from AllCells[7], and PBMCs form a diseased Acute Lymphoblastic Leukemia donor obtained by 10x Genomics from Sanguine Biosciences[8].

In order to analyse the datasets together with `tagtango`, we first independently processed and annotated each of them. To do so, we followed a three-step process: first, we down-

loaded the corresponding h5 file from the 10x Genomics platform; we then processed each dataset using the R packages 'Seurat'[10], 'SingleCellExperiment'[2] and 'scater'[14]; and we use the R packages 'celldex' and 'SingleR'[3] to annotate the single-cell datasets, identifying the main cell types. In R, the processing of each dataset is as follows:

```
# Load libraries
library(Seurat)
library(SingleCellExperiment)
library(scater)
library(SingleR)
library(celldex)

# Set path to the h5 file downloaded from www.10xgenomics.com
path <- "path_to_h5_file"

# Load raw data
sce <- as.SingleCellExperiment(
        CreateSeuratObject(
             Seurat::Read10X_h5(path)
        )
  )

# calculate the proportion of mitochondrial reads
mt.genes <- rownames(sce)[grep("^MT-",rownames(sce))]
sce <- addPerCellQC(sce, subsets = list(Mito = mt.genes))

# perform QC
qc.lib <- isOutlier(sce$sum, log=TRUE, type="lower")
qc.nexprs <- isOutlier(sce$detected, log=TRUE, type="lower")
qc.mito <- isOutlier(sce$subsets_Mito_percent, type="higher")
sce$discard <- qc.lib | qc.nexprs | qc.mito
sce <- sce[,!sce$discard]

# Normalize counts
sce <- computeLibraryFactors(sce)
sce <- logNormCounts(sce)

# Annotate dataset with SingleR
```

```
ref <- BlueprintEncodeData()
pred <- SingleR(test=sce, ref=ref, labels=ref$label.main)
colData(sce) <- cbind(colData(sce), Main.labels = pred$labels)
```

This workflow provided us with two annotated `SingleCellExperiment`, one for the healthy patient (i.e. `sce_healthy`) and one for the cancer patient (i.e. `sce_cancer`). The last step before analysing the files with `tagtango` was to integrate these objects, removing biologically irrelevant batch effects. To do so, we used packages ‘scran’[12] and ‘batchelor’[9] to correct the log-expression values via linear regression:

```
# Load libraries
library(scran)
library(batchelor)

# Find genes in common
universe <- intersect(rownames(sce_cancer_clean), rownames(sce_healthy_
   clean))

# Variance model
dec_cancer_clean <- modelGeneVarByPoisson(sce_cancer)[universe,]
dec_healthy_clean <- modelGeneVarByPoisson(sce_healthy)[universe,]

# Find HVGs
combined.dec <- combineVar(dec_cancer_clean, dec_healthy_clean)
chosen.hvgs <- combined.dec$bio > 0

# Per-batch scaling normalization
rescaled <- multiBatchNorm(sce_cancer[universe,], sce_healthy[universe,])
pbmc_cancer <- rescaled[[1]]
pbmc_healthy <- rescaled[[2]]

# Merge datasets and calculate TSNA
rescaled <- rescaleBatches(pbmc_cancer, pbmc_healthy)
rescaled <- runPCA(rescaled, subset_row=chosen.hvgs,
                   exprs_values="corrected",
                   BSPARAM=BiocSingular::RandomParam())
rescaled <- runTSNE(rescaled, dimred="PCA")
```

```
# Copy annotations and batch information
rescaled$batch <- factor(rescaled$batch, levels = c(1,2), labels = c("
   cancer", "healthy"))
rescaled$annotations <- c(pbmc_cancer$Main.labels, pbmc_healthy$Main.labels
   )
```

Using `tagtango`, we then compared the batch information and the annotations found with 'singleR' (Supplementary Figure **S3**). In particular, we focused on the differences across cells annotated as CD4+ T-cells across the two datasets. Again, `tagtango` highlighted differences in the normalized expression of several interesting genes, all related to immune response in cancer. Notably, JUNB has been identified in the past as a gatekeeper for a certain type of lymphoid leukemia[16], and LTB has been shown to promote the development of T-cell acute lymphoblastic leukemia[5]. Likewise, genes such as CD27 or IL-32 have been closely associated to the clinical outcome and prognostic of acute lymphoblastic leukemia patients[1,4,19], and there are links between the expression of IL-7R and TCF7 with subsets of these patients[15,20]. Overall, `tagtango` efficiently identified genes associated with lymphocyte development, transcriptional regulation, and immune signaling pathways in patients with acute lymphoblastic leukemia. However, further work would be required to validate these findings and ensure their biological relevance.

Supplementary References

[1] Abobakr, A., Osman, R. A., Kamal, M. A. M., Abdelhameed, S. A., Ismail, H. H., Kamel, M. M., Alsharif, K. F. & Hamad, N. R. (2023). Clinical and prognostic significance of CD27 and CD44 expression patterns in Egyptian pediatric patients with B-precursor acute lymphoblastic leukemia. *Hematology, Transfusion and Cell Therapy*, S2531–1379(23)02592–0.

[2] Amezquita, R. A., Lun, A. T. L., Becht, E., Carey, V. J., Carpp, L. N., Geistlinger, L., Marini, F., Rue-Albrecht, K., Risso, D., Soneson, C., Waldron, L., Pagès, H., Smith, M. L., Huber, W., Morgan, M., Gottardo, R. & Hicks, S. C. (2020). Orchestrating single-cell analysis with Bioconductor. *Nature Methods*, 17, 137–145.

[3] Aran, D., Looney, A. P., Liu, L., Wu, E., Fong, V., Hsu, A., Chak, S., Naikawadi, R. P.,

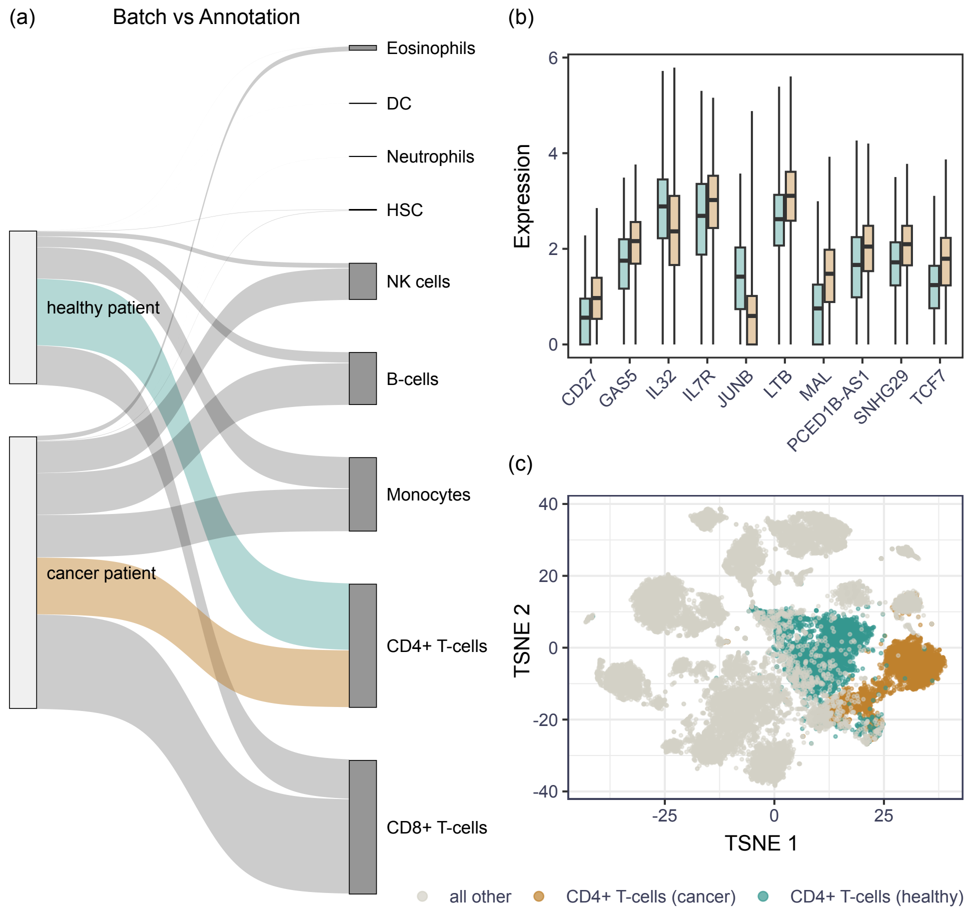

Supplementary Figure S3: Overview of the comparison between datasets using `tagtango`. Panel (a) displays a Sankey diagram comparing the batch information and main cell types. The coloured links in the diagram indicate the cell populations selected for deeper analysis. Panel (b) displays a direct comparison of the normalized and batch-corrected RNA expression for the two selected cell populations, including only markers selected as relevant. The colours of the bars match those of the selected links in panel (a). Panel (c) presents the TSNE representation of the RNA expression, where the colours of the points match those of the selected links in panel (a).

Wolters, P. J., Abate, A. R., Butte, A. J. & Bhattacharya, M. (2019). Reference-based analysis of lung single-cell sequencing reveals a transitional profibrotic macrophage. *Nature Immunology*, 20, 163–172.

[4] Chen, D., Gerasimčik, N., Camponeschi, A., Tan, Y., Wu, Q., Brynjolfsson, S., Zheng, J., Abrahamsson, J., Nordlund, J., Lönnerholm, G., Fogelstrand, L. & Mårtensson, I.-L. (2017). CD27 expression and its association with clinical outcome in children and adults with pro-B acute lymphoblastic leukemia. *Blood Cancer Journal*, 7, e575–e575.

[5] Fernandes, M. T., Ghezzo, M. N., Silveira, A. B., Kalathur, R. K., Póvoa, V., Ribeiro,

A. R., Brandalise, S. R., Dejardin, E., Alves, N. L., Ghysdael, J., Barata, J. T., Yunes, J. A. & dos Santos, N. R. (2015). Lymphotoxin-β receptor in microenvironmental cells promotes the development of T-cell acute lymphoblastic leukaemia with cortical/mature immunophenotype. *British Journal of Haematology*, 171, 736–751.

[6] Genomics, x. (2018). 10k PBMCs from a Healthy Donor, Single Cell Gene Expression Dataset by Cell Ranger 3.0.0.

[7] Genomics, x. (2021). 10k Human PBMCs, 5' v2.0, Chromium X, Single Cell Immune Profiling dataset analyzed using Cell Ranger 6.1.0.

[8] Genomics, x. (2024). 10k Human Diseased PBMCs (ALL) Freshly Processed, Single Cell Immune Profiling dataset analyzed using Cell Ranger 8.0.1.

[9] Haghverdi, L., Lun, A. T. L., Morgan, M. D. & Marioni, J. C. (2018). Batch effects in single-cell RNA-sequencing data are corrected by matching mutual nearest neighbors. *Nature Biotechnology*, 36, 421–427.

[10] Hao, Y., Stuart, T., Kowalski, M. H., Choudhary, S., Hoffman, P., Hartman, A., Srivastava, A., Molla, G., Madad, S., Fernandez-Granda, C. & Satija, R. (2023). Dictionary learning for integrative, multimodal and scalable single-cell analysis. *Nature Biotechnology*.

[11] L. Lun, A. T., Bach, K. & Marioni, J. C. (2016). Pooling across cells to normalize single-cell RNA sequencing data with many zero counts. *Genome Biology*, 17, 75.

[12] Lun, A. T. L., McCarthy, D. J. & Marioni, J. C. (2016). A step-by-step workflow for low-level analysis of single-cell RNA-seq data with Bioconductor.

[13] Maynard, K. R., Collado-Torres, L., Weber, L. M., Uytingco, C., Barry, B. K., Williams, S. R., Catallini, J. L., Tran, M. N., Besich, Z., Tippani, M., Chew, J., Yin, Y., Kleinman, J. E., Hyde, T. M., Rao, N., Hicks, S. C., Martinowich, K. & Jaffe, A. E. (2021). Transcriptome-scale spatial gene expression in the human dorsolateral prefrontal cortex. *Nature Neuroscience*, 24, 425–436.

[14] McCarthy, D. J., Campbell, K. R., Lun, A. T. L. & Wills, Q. F. (2017). Scater:

Pre-processing, quality control, normalization and visualization of single-cell RNA-seq data in R. *Bioinformatics (Oxford, England)*, 33, 1179–1186.

[15] Oliveira, M. L., Akkapeddi, P., Ribeiro, D., Melão, A. & Barata, J. T. (2019). IL-7R-mediated signaling in T-cell acute lymphoblastic leukemia: An update. *Advances in Biological Regulation*, 71, 88–96.

[16] Ott, R. G., Simma, O., Kollmann, K., Weisz, E., Zebedin, E. M., Schorpp-Kistner, M., Heller, G., Zöchbauer, S., Wagner, E. F., Freissmuth, M. & Sexl, V. (2007). JunB is a gatekeeper for B-lymphoid leukemia. *Oncogene*, 26, 4863–4871.

[17] Pardo, B., Spangler, A., Weber, L. M., Page, S. C., Hicks, S. C., Jaffe, A. E., Martinowich, K., Maynard, K. R. & Collado-Torres, L. (2022). spatialLIBD: An R/Bioconductor package to visualize spatially-resolved transcriptomics data. *BMC Genomics*, 23, 434.

[18] Ramos, M., Schiffer, L., Re, A., Azhar, R., Basunia, A., Rodriguez, C., Chan, T., Chapman, P., Davis, S. R., Gomez-Cabrero, D., Culhane, A. C., Haibe-Kains, B., Hansen, K. D., Kodali, H., Louis, M. S., Mer, A. S., Riester, M., Morgan, M., Carey, V. & Waldron, L. (2017). Software for the Integration of Multiomics Experiments in Bioconductor. *Cancer Research*, 77, e39–e42.

[19] Shim, S., Lee, S., Hisham, Y., Kim, S., Nguyen, T. T., Taitt, A. S., Hwang, J., Jhun, H., Park, H.-Y., Lee, Y., Yeom, S. C., Kim, S.-Y., Kim, Y.-G. & Kim, S. (2022). A Paradoxical Effect of Interleukin-32 Isoforms on Cancer. *Frontiers in Immunology*, 13.

[20] Van Thillo, Q., De Bie, J., Seneviratne, J. A., Demeyer, S., Omari, S., Balachandran, A., Zhai, V., Tam, W. L., Sweron, B., Geerdens, E., Gielen, O., Provost, S., Segers, H., Boeckx, N., Marshall, G. M., Cheung, B. B., Isobe, K., Kato, I., Takita, J., Amos, T. G., Deveson, I. W., McCalmont, H., Lock, R. B., Oxley, E. P., Garwood, M. M., Dickins, R. A., Uyttebroeck, A., Carter, D. R., Cools, J. & de Bock, C. E. (2021). Oncogenic cooperation between TCF7-SPI1 and NRAS(G12D) requires β-catenin activity to drive T-cell acute lymphoblastic leukemia. *Nature Communications*, 12, 4164.

[21] Zeng, H., Shen, E. H., Hohmann, J. G., Oh, S. W., Bernard, A., Royall, J. J., Glattfelder, K. J., Sunkin, S. M., Morris, J. A., Guillozet-Bongaarts, A. L., Smith, K. A., Ebbert, A. J., Swanson, B., Kuan, L., Page, D. T., Overly, C. C., Lein, E. S., Hawrylycz, M. J., Hof, P. R., Hyde, T. M., Kleinman, J. E. & Jones, A. R. (2012). Large-Scale Cellular-Resolution Gene Profiling in Human Neocortex Reveals Species-Specific Molecular Signatures. *Cell*, 149, 483–496.
